# Patients’ and Publics’ Preferences for Data-Intensive Health Research Governance: Survey Study

**DOI:** 10.2196/36797

**Published:** 2022-09-07

**Authors:** Sam H A Muller, Ghislaine J M W van Thiel, Marilena Vrana, Menno Mostert, Johannes J M van Delden

**Affiliations:** 1 Department of Medical Humanities Julius Center for Health Sciences and Primary Care University Medical Center Utrecht, Utrecht University Utrecht Netherlands; 2 European Heart Network Brussels Belgium

**Keywords:** data-intensive health research, big data, data sharing, patient and public preferences, health data sharing conditions, ethics, governance, policy, patient and public involvement, research participants, trust

## Abstract

**Background:**

Patients and publics are generally positive about data-intensive health research. However, conditions need to be fulfilled for their support. Ensuring confidentiality, security, and privacy of patients’ health data is pivotal. Patients and publics have concerns about secondary use of data by commercial parties and the risk of data misuse, reasons for which they favor personal control of their data. Yet, the potential of public benefit highlights the potential of building trust to attenuate these perceptions of harm and risk. Nevertheless, empirical evidence on how conditions for support of data-intensive health research can be operationalized to that end remains scant.

**Objective:**

This study aims to inform efforts to design governance frameworks for data-intensive health research, by gaining insight into the preferences of patients and publics for governance policies and measures.

**Methods:**

We distributed a digital questionnaire among a purposive sample of patients and publics. Data were analyzed using descriptive statistics and nonparametric inferential statistics to compare group differences and explore associations between policy preferences.

**Results:**

Study participants (N=987) strongly favored sharing their health data for scientific health research. Personal decision-making about which research projects health data are shared with (346/980, 35.3%), which researchers/organizations can have access (380/978, 38.9%), and the provision of information (458/981, 46.7%) were found highly important. Health data–sharing policies strengthening direct personal control, like being able to decide under which conditions health data are shared (538/969, 55.5%), were found highly important. Policies strengthening collective governance, like reliability checks (805/967, 83.2%) and security safeguards (787/976, 80.6%), were also found highly important. Further analysis revealed that participants willing to share health data, to a lesser extent, demanded policies strengthening direct personal control than participants who were reluctant to share health data. This was the case for the option to have health data deleted at any time (*P*<.001) and the ability to decide the conditions under which health data can be shared (*P*<.001). Overall, policies and measures enforcing conditions for support at the collective level of governance, like having an independent committee to evaluate requests for access to health data (*P*=.02), were most strongly favored. This also applied to participants who explicitly stressed that it was important to be able to decide the conditions under which health data can be shared, for instance, whether sanctions on data misuse are in place (*P*=.03).

**Conclusions:**

This study revealed that both a positive attitude toward health data sharing and demand for personal decision-making abilities were associated with policies and measures strengthening control at the collective level of governance. We recommend pursuing the development of this type of governance policy. More importantly, further study is required to understand how governance policies and measures can contribute to the trustworthiness of data-intensive health research.

## Introduction

Various proposals exist for an ethical governance framework for data-intensive health research [1,2]. However, lessons learned point out that no fit-for-purpose governance framework currently exists [3,4]. At the same time, oversight in large, big data–driven research projects cannot be achieved by simply collecting and synthesizing existing governance elements of databases that participate in the project [3,5]. In addition, there is growing awareness of the need for a so-called social license to ensure patients’ and publics’ support, cooperation, and trust with data-intensive health research [5-8]. These governance challenges are echoed in the current preparatory developments of a European Health Data Space, which is part of the European Strategy for Data [9-11]. Therefore, it has become evident that we need to understand patients’ and publics’ preferences about what such a governance framework should look like. In this context, we use the plural “publics” to stress the diversity contained within the singular “public at large” [12,13].

Previous empirical research on patients’ and publics’ views revealed that they are generally positive about data sharing. Nonetheless, their support for data-intensive health research is not unconditional [5,8,14]. Moreover, when conditions are met, people tend to be more supportive of data sharing for data-intensive health research [5,8,14-18]. Protecting patients’ privacy and safeguarding confidentiality and security of personal health data are important conditions for support of data sharing [5,8,14,19,20]. The possible risk of harm induced by health data research plays an important role in these conditions and impacts overall support in the long run [5,8,14,18]. More specifically, the possibility of abuse or misuse of data carries important weight in patients’ risks perceptions [5,8,14,17-19,21]. Whereas the status of data-intensive health research as a common good that contributes to public benefit is widespread, this status needs to be guaranteed to gain and retain support [8,15,22,23]. In light of this, empirical findings point out that it is crucial to strike an appropriate balance between benefits and risks across the stakeholders involved in health data research [6,14,18,24,25]. Secondary use by commercial parties, such as pharmaceutical companies, complicates maintaining such a balance. Commercial involvement is considered to be accompanied by motivations that severely diminish the perceived public benefit of data use, such as profit-seeking [14,18,19]. Therefore, personal control plays an important role in patients’ and publics’ views on governance [8,17,18], such as requirements for specific informed consent [20,22,26,27]. Yet, the feasibility of specific informed consent within the context of data-intensive health research has been questioned [8,18]. Alternatives in which ethics and governance frameworks warrant trust in different ways are increasingly seen as viable and appropriate [6,28]. One example is to entrust data access committees with a more prominent role in controlling data sharing and research [19,22,23]. Still, low levels of awareness and understanding of research, oversight, and governance practices by laypeople currently form obstacles to pursuing this path [8,14,17]. As the distance between research and patients and publics increases [5,17-19], seeking transparent and engaged forms of communication is pivotal [8,12]. Moreover, relevant information regarding the context of data use, particularly *by whom,* and the content of research should be provided [1,20,23,29].

The notion of personal control still plays an important role in patients’ and publics’ attitudes to data-intensive health research governance [8,17,18]. However, empirical research increasingly points to governance as a means of garnering trust in research, research organizations, and data-sharing practices [5,6,17,18]. The empirical literature reveals some valuable insights about conditions that are important for patients’ and publics’ support for data-intensive health research. Yet, much less is known about which types of governance policies and measures are desired [5]. In this study, we build upon these insights as well as prior conceptual work on elements for socially sanctioned governance [5], to further operationalize governance and seek empirical input by asking patients and publics. Therefore, the aim of this study was to gain further insight regarding how conditions for support of governance can be put into practice in a governance framework. Accordingly, we used a structure involving 3 themes: (1) views on conditions for health data sharing, (2) preferences for health data sharing policies and governance measures, and (3) the role and implementation of patient and public involvement.

## Methods

### Aim and Design

The aim of this survey was to establish patients’ and publics’ preferences for data-intensive health research governance. The first version of the questionnaire was pilot tested twice with patient panels from the European Heart Network and its Dutch member organization Harteraad. Following this, minor changes were made to phrasing of the questions. The final questionnaire consisted of 17 questions distributed over 5 pages, taking respondents approximately 10 minutes to complete. Respondents were able to review and change their answers. In addition to the English version, the questionnaire was translated to Danish, German, French, Dutch, Swedish, Finnish, Spanish, Portuguese, Romanian, and Slovenian to make it easier for people to participate and increase widespread uptake. We used 5-point, Likert-item questions as well as multiple-choice questions (see Multimedia Appendix 1). Duplicate entries were avoided by using cookies that expired after 6 months, preventing users from accessing the survey twice.

### Ethical Considerations

Approval from an ethical committee was not necessary for this type of unobtrusive, nonmedical scientific research. Under Dutch law, this research is exempt from review by a medical research ethics committee (Medical Research Involving Human Subjects Act [WMO]; Central Committee on Research Involving Human Subjects). Participants gave their informed consent for the use of their answers for scientific research prior to the start of the questionnaire.

### Setting

The survey was conducted online among a purposive sample by digital distribution via the European Heart Network, a partner of the BigData@Heart project. The European Heart Network is a European alliance of foundations and associations dedicated to preventing cardiovascular diseases, supporting and representing patient interests throughout Europe. Distribution was facilitated by the European Heart Network itself, its 27 member organizations, and its participation in the European Commission’s Health Policy Platform. The survey was distributed via patient panels; email and online newsletters; and calls for participation in web items, email, and various social media platforms (Twitter, Facebook, LinkedIn). The survey was administered using the Qualtrics XM survey tool. The only inclusion criterion was age 18 years and older. Participation was voluntary and without incentives. The survey was accessible from February 9, 2021, until May 10, 2021.

### Analysis

We analyzed both complete and incomplete questionnaires. Analysis focused on descriptive statistics and exploring patterns within and between the thematized variables. Data were analyzed using SPSS version 26 (IBM Corp, Armonk, NY). For Likert-item ordinal variables, we report descriptive statistics including response percentages for each category, the median, and IQR. For multiple-choice categorical variables, we report frequencies and percentages for each category as well as the mode. For the descriptive statistics, we report valid percentages.

Using inferential statistics, we compared groups and tested for associations between preferences for data sharing conditions as well as policies and measures. We employed nonparametric chi-square tests of independence, Mann-Whitney *U* tests, Kruskal-Wallis tests, and Spearman rank order correlations since the assumptions underlying parametric statistics were violated. More fundamentally, nonparametric statistics were more appropriate due to the ordinal and categorical levels of measurement of the survey variables [30-33].

We used an α level of .05 to determine significance for all statistical tests. All tests were 2-tailed. For all tests reported, the (nonparametric) assumptions were met. Since missing data were diffuse, specific missing data patterns were not apparent, and <5% of data were missing for all variables, missing data have been assumed ignorable. We treated missing data in the nonparametric statistical tests via customary pairwise deletion of cases, which is robust for large sample sizes with diffuse and small amounts of missing data [34,35]. As a result, sample sizes varied slightly across tests. To infer the direction of associations for chi-square tests of independence, dependent variables were dummy coded.

## Results

A total of 987 respondents took part in the survey: 81.7% (788/964) of the respondents identified as being cardiovascular disease patients, and 58.9% (576/978) of the respondents were male. Respondents were relatively old, and 80.5% (782/972) of the respondents came from the Netherlands. See Table 1 for an overview of the background variables.

**Table 1:** Frequencies of background variables (n=987).

Variables			Results, n (%)^a^
**Gender^b^**			
	Female	400 (40.9)	
	Male	576 (58.9)	
	Other	2 (0.2)	
**Age range (years)^b^**			
	18-30	25 (2.6)	
	31-40	32 (3.3)	
	41-50	83 (8.5)	
	51-60	188 (19.2)	
	61-70	339 (34.7)	
	≥71	311 (31.8)	
**Country of residence^c,d^**			
	Belgium	9 (0.9)	
	Finland	10 (1.0)	
	Germany	68 (7.0)	
	Ireland	5 (0.5)	
	Netherlands	782 (80.5)	
	Portugal	7 (0.7)	
	Sweden	7 (0.7)	
	United Kingdom	68 (7.0)	
**Education level^e^**			
	Less than secondary/high school	18 (1.9)	
	Secondary/high school	212 (21.9)	
	Vocational/professional qualifications	343 (35.4)	
	Bachelor degree	168 (17.4)	
	Master degree	146 (15.1)	
	Postgraduate degree	58 (6.0)	
	Other	23 (2.4)	
**Identification as a patient^f^**			
	Yes	788 (81.7)	
	No	176 (18.3)	

^a^Percentages given are valid percentages; n varies per variable.

^b^n=978.

^c^Countries with a percentage >0.5% are shown.

^d^n=972.

^e^n=968.

^f^n=964.

### Views on Conditions for Health Data Sharing

We asked participants about their general attitudes to sharing their health data for scientific health research purposes. Generally, 62.7% (615/981; median 5, IQR 4-5) of participants in the survey indicated they *strongly favored* sharing their health data for health research. This was followed by 23.8% (233/981) who *somewhat favored* health data sharing. A total of 86.5% (848/981) was in favor of sharing their health data. Several aspects of conditions for health data sharing were considered important. Respondents (458/981, 46.7%; median 5, IQR 3-5) found receiving information about research projects *highly important*, while 38.9% (380/978; median 5, IQR 2-5) of the respondents found it *highly important* to be able to decide which researchers or organizations have access to their data. Moreover, 35.3% (346/980; median 5, IQR 2-5) saw being able to decide which research projects had access to their data as *highly important*, and 23.1% (226/980) found this *highly unimportant.* Choosing which types of health data are shared was considered *highly important* by 33.3% (325/977; median 5, IQR 1-5) of respondents. Conversely, 26.7% (261/977) indicated this was *highly unimportant* to them.

Sharing data anonymously was preferred by 33.3% (328/985; mode 2), whereas 22.9% (226/985) indicated anonymity should be required. Pseudonymous data sharing was preferred by 26.1% (257/985) of the respondents. Respondents indicated that researchers or organizations having a relevant research question (423/983, 43.0%; mode 1) and researchers from government or not-for-profit organizations (423/983, 43.0%) should have access to their data. See Table S1 in Multimedia Appendix 2 for the detailed descriptive results.

We tested whether the background variables of age, gender, education level, and identification as a patient were associated with participants’ willingness to share their health data for health research (see Tables 2 and 3). Higher education levels were significantly, positively associated with higher levels of willingness to share health data (ρ=0.096, n*=*962, *P=*.003). However, all education levels strongly favored sharing their health data (median 5), except for those with less than a secondary or high school education. We therefore additionally tested the dependent variables for associations with education level.

**Table 2:** Association between background variables and willingness to share health data, assessed using the Kruskal-Wallis test and Spearman rank order correlation.

Variables			Statistic		*P* value
**Kruskal-Wallis test (n=972)**					
	Age	χ^2^_5_=4.2		.52	
	Gender	χ^2^_2_=2.5		.28	
**Spearman rank order correlation (n=962)**					
	Education level	ρ=0.096		.003	

**Table 3:** Association between background variables and willingness to share health data, assessed using the Mann-Whitney *U* test.

Variable			Median		Mean rank		*U*		*z*		*P* value
**Identification as a patient (n=958)**											
	Yes (n=784)	5		486		62914		–1.87		.06	
	No (n=174)	5		449							

Participants’ willingness to share health data was significantly associated with anonymity preferences (*χ*^2^_12_=134.5, n*=*979, *P<*.001, φ*_c_*=0.214); 32.9% (279/848) of participants in favor of health data sharing preferred anonymity, followed by 28.2% (239/848) of participants who preferred pseudonymization. In contrast, 72% (31/43) of participants who opposed health data sharing required anonymity. Participants’ level of education was also significantly associated with preferences for anonymity (*χ*^2^_18_=44.5, n*=*966, *P<*.001, φ*_c_*=0.124). However, preferences for anonymity followed the same pattern across education levels, as with willingness to share health data.

We found that respondents who are more willing to share data are less interested in choosing which of their health data are shared, for which projects, and with whom (see Table 4).

In addition, willingness to share health data was significantly associated with which types of researchers should have access to participants’ data (*χ*^2^_12_=34.9, n*=*977, *P<*.001, φ*_c_*=0.109); 43.2% (366/847) of those favoring health data sharing preferred access to their data by all researchers and organizations with a relevant research question. Slightly less (355/847, 41.9%) wanted only researchers from government or not-for-profit organizations to have access to their data.

**Table 4:** Spearman rank order correlations (ρ) between willingness to share health data and views on conditions for health data sharing.

Variable			1. In general, how do you feel about sharing your health data for health research?		2. How important is it that you can decide for which research projects your health data are shared?		3. How important is it that you are informed about the research projects for which your health data is shared?		4. How important is it that you can decide for yourself which researchers/organizations your health data is shared with?		5. How important is it that you can choose which health data is shared and which is not?
**1. In general, how do you feel about sharing your health data for health research?**											
	ρ	1		–0.187		–0.034		–0.179		–0.276	
	*P* value	—^a^		<.001		.28		<.001		<.001	
**2. How important is it that you can decide for which research projects your health data are shared?**											
	ρ	–0.187		1		0.539		0.634		0.638	
	*P* value	<.001		—^a^		<.001		<.001		<.001	
**3. How important is it that you are informed about the research projects for which your health data is shared?**											
	ρ	–0.034		0.539		1		0.555		0.482	
	*P* value	.28		<.001		—^a^		<.001		<.001	
**4. How important is it that you can decide for yourself which researchers/organizations your health data is shared with?**											
	ρ	–0.179		0.634		0.555		1		0.713	
	*P* value	<.001		<.001		<.001		—^a^		<.001	
**5. How important is it that you can choose which health data is shared and which is not?**											
	ρ	–0.276		0.638		0.482		0.713		1	
	*P* value	<.001		<.001		<.001		<.001		—^a^	

^a^Not applicable.

### Preferences for Health Data Sharing Policies and Governance Measures

Study participants expressed their views on data sharing policies and governance measures for researchers sharing or using health data from databases: 80.6% (787/976) considered it *highly important* that databases are highly secure and difficult to get into (median 5, IQR 5-5). The possibility to have health data deleted at any time was considered *highly important* by 60.6% (589/972; median 5, IQR 4-5) of participants, while 55.5% (538/969; median 5, IQR 4-5) deemed it *highly important* to be able to decide on conditions for health data sharing, such as limitations for international data sharing or commercial use. Last, researcher reliability checks before gaining data access were judged *highly important* by 83.2% (805/967; median 5, IQR 5-5) of participants.

Moreover, we asked participants which 3 of 7 governance measures they favored most. Having sanctions for data misuse was chosen most often by 23.5% (637/2708; mode 6) of the participants. Also, 22.4% (607/2708) favored having data access requests evaluated by an independent data access committee. See Table S2 in Multimedia Appendix 2 for the detailed descriptive results.

Being more willing to share health data was associated with 2 data sharing policies (see Table S3 in Multimedia Appendix 2). The possibility to have health data deleted at any time (ρ=–0.118, n*=*967, *P<*.001) and being able to decide on conditions under which health data can be shared (ρ=–0.173, n*=*964, *P<*.001) were significantly associated with being less willing to share health data. In addition, higher education levels were significantly associated with greater preference for database security (ρ=0.145, n*=*957, *P<*.001).

Willingness to share health data was significantly associated with several data sharing governance measures: 63.4% (538/848) of participants who favored sharing health data favored an independent committee to evaluate health data access requests (*χ*^2^_4_=11.8, n*=*981, *P=*.02, φ*_c_*=0.110). Also, 66.2% (561/848) of those favoring health data sharing preferred subjecting those who misuse data to sanctions (*χ*^2^_4_=7.9, n*=*981, *P=*.096, φ*_c_*=0.090). Conversely, 70.0% (594/848) of participants in favor of health data sharing did not deem it important that researchers should ask for consent each time data are used (*χ*^2^_4_=26.4, n*=*981, *P<*.001, φ*_c_*=0.164). Obtaining approval from representatives on behalf of patients to use their data was not preferred by 74.8% (634/848) of participants in favor of health data sharing (*χ*^2^_4_=10.4, n*=*981, *P=*.03, φ*_c_*=0.103). Additionally, education level was significantly associated with preference for having an independent committee for data access requests (*χ*^2^_6_=26.9, n*=*968, *P<*.001, φ*_c_*=0.167). Notifying patients and citizens that their health data will be re-used was also significantly related with education level (*χ*^2^_6_=19.5, n*=*968, *P=*.003, φ*_c_*=0.142).

We furthermore found that being able to decide on conditions under which data can be shared was positively and strongly associated with the possibility of having health data deleted at any time. A highly secure database and researcher reliability checks before data access were also strongly related with being able to decide on conditions under which health data can be shared (see Table S4 in Multimedia Appendix 2). Moreover, 62.4% (455/729) of those preferring to decide on data sharing conditions favored sanctions for misuse. However, informing patients or citizens about results of research studies that used their health data was not judged important by 81.6% (595/729) of participants. Similarly, allowing researchers to use health data only for a pre-approved period of time was not deemed important by 63.5% (463/729) of participants. Asking consent each time data are used (450/729, 61.7%) and notifying patients of reuse (441/729, 60.5%) were also not considered important by those preferring to decide on data sharing conditions. See Table 5 for a detailed overview.

**Table 5:** Chi-square tests of independence for association between preference to decide on conditions and health data sharing governance measures (n=969).

Chi-square test of independence	Chi-square (*df*)	*P* value	Cramér V	Moderately/slightly important, n (%)
Requests for access to health data should be evaluated by an independent (data access) committee (1)	7.2 (4)	.14	0.086	445 (61.0)
Researchers should ask for consent of the patients/citizens from whom these data originate each time their health data will be used (2)	65.6 (4)	<.001	0.260	279 (38.3)
Researchers should notify patients/citizens that their health data will be re-used (3)	14.8 (4)	.005	0.124	288 (39.5)
Researchers should obtain approval from representatives on behalf of patients/citizens to use their health data (4)	11.8 (4)	.02	0.111	161 (22.1)
Researchers should only be allowed to use the health data for a pre-approved period of time. After this period, the health data can no longer be used (5)	16.6 (4)	.002	0.131	266 (36.5)
If health data is misused, those concerned must be subject to sanctions (6)	10.5 (4)	.03	0.104	455 (62.4)
Researchers should only inform patients/citizens about the results of the research studies for which their health data was used (7)	15.1 (4)	.005	0.125	134 (18.4)

### The Role of Patient and Public Involvement in Health Data Sharing

We asked participants about their opinion on patient participation, which we defined as research conducted by talking to, rather than about, patients. About one-half (466/987, 47.2%; mode 1) of the respondents had ever heard of patients being involved in health research. A smaller group had ever participated in patient and public involvement activities, such as participation in review committees or sounding board groups (214/987, 21.7%; mode 2). Most of the participants considered each patient and public involvement role in health data research important. Specifically, 40.3% (383/951; median 4, IQR 3-5) deemed it *fairly important* that patients and publics are involved in choices about consent and providing information about health data use, and 31.4% (299/951) deemed this *extremely important*. Involvement in evaluating health data sharing requests was considered *fairly important* by 39.3% (362/921; median 4, IQR 3-4) and *extremely important* by 21.4% (197/921) of participants. Involvement in choices about which research questions are relevant in medical science was judged *fairly important* by 38.8% (361/931; median 4, IQR 2-4), while 35.4% (330/931; median 4, IQR 2-4) thought so about making choices about how to conduct research using health data. Slightly fewer (292/949, 30.8%; median 4, IQR 3-4) considered patient and public involvement in choices about disseminating research results *fairly important*. See Table S5 in Multimedia Appendix 2 for the detailed descriptive results.

Awareness of and having participated in patient and public involvement activities were significantly related with greater willingness to share health data (see Table 6). In addition, respondents with (less than) secondary or high school education were significantly less aware of patient and public involvement (*χ*^2^_12_=52.1, n*=*968, *P<*.001, φ*_c_*=0.164).

Low willingness to share health data was significantly associated with greater importance for 3 patient and public involvement roles (see Table S6 in Multimedia Appendix 2). The importance of involvement in making choices about consent and providing information decreased when health data sharing was favored. We observed the same decrease in importance for involvement in evaluating data sharing requests and the dissemination of research results. Additionally, lower education levels were significantly related with greater importance of involvement in making choices about research questions (ρ=–0.103, n*=*913, *P=*.002) and how to conduct health data research (ρ=–0.148, n*=*193, *P<*.001). Patient and public involvement in evaluating health data sharing requests (ρ=–0.089, n*=*903, *P=*.007) and disseminating research results (ρ=–0.189, n*=*931, *P<*.001) was also considered more important by those with lower education levels. What is more, the importance of patient and public involvement roles was significantly greater for participants who had ever heard of patients and publics being involved in health research. This was not the case for involvement in disseminating research results (see Table S7 in Multimedia Appendix 2).

**Table 6:** Association between willingness to share health data and awareness of and having participated in patient and public involvement activities, assessed using a Mann-Whitney *U* test.

Variable			Median		Mean rank		*U*		*z*		*P* value
**Awareness of patient and public involvement (n=902)**											
	Yes (n=462)	5		471		92429		–2.75		.006	
	No (n=440)	5		431							
**Having participated in patient and public involvement activities (n=905)**											
	Yes (n=212)	5		507		71603		–2.19		.03	
	No (n=738)	5		467							

## Discussion

### Principal Findings

In this survey of patients’ and publics’ views about data-intensive health research governance, respondents were very much in favor of sharing their health data for scientific health research. Nevertheless, in correspondence with the literature [8,14], our findings indicate that support for data-intensive health research is not unconditional. People require additional means of exercising control. Control is desired at the individual level as well as at the collective level of governance in the form of various policies and measures.

In terms of privacy, anonymous data sharing was preferred most, whereas it was required far less. Instead, pseudonymous health data sharing was favored to a greater extent. Further analysis revealed that those favoring health data sharing had a more lenient stance on anonymity. Conversely, an overwhelming majority of those opposing health data sharing considered anonymity to be a requirement. This is in line with previous research, which indicates that anonymization is an important factor for support of health data sharing governance [15,20,36]. De-identification is important for privacy but also functions as a form of data security at large. However, there are different views on what would be feasible and desirable approaches to implement de-identification in practice [4]. Moreover, what should be “default” for safeguarding confidentiality and health data security remains a topic of discussion [8,36,37]. By pointing out the acceptability and desirability of pseudonymity, our findings provide further input in this debate. Our findings dispute that anonymization still forms a salient approach in the discussion around data de-identification. Moreover, this highlights the importance of exploring both technical and legal possibilities in practice, so that pseudonymous data sharing can be pursued as a way forward for researching health data [38,39].

Participants in our study were of the opinion that all researchers or organizations having a relevant research question should have access to their data. This goes against restricting data access to public or not-for-profit researchers or organizations, which is commonly preferred. Moreover, our findings point out that those opposing health data sharing preferred access by public or not-for-profit researchers or organizations only to a far greater extent. This corroborates that pursuing collective or public benefit leads to greater support for health data sharing and research [8,22,29]. In particular, our findings specify how providing warranties can contribute to maintaining support. Foremost, warranties of public benefit need not necessarily be limited to health data access by government, public, or not-for-profit researchers only. Rather, more attention should be paid to how the relevance of research purposes can be explicated in such a way that conditions for support of health data sharing are fulfilled. At its core, this necessitates researchers and participants to articulate together what makes research purposes relevant in the first place.

Our findings confirm that private use of shared health data is detrimental to support and willingness to partake in data-intensive health research, as it is often accompanied by a profit motive. This confirms previous links between commercial involvement and motivations for data use that were seen as undesirable [8,14,17-19]. As public-private cooperation increases in data-intensive health research, rebalance should be sought by addressing the social relevance of research questions for patients and publics. Ascertaining what contributes to the relevance of research questions and practices from the perspective of patients and publics would provide a promising way forward.

We distinguish 2 types of policies and measures that are considered important in relation to governing data-intensive health research. First, at the individual-level, personal control over participants’ health data is strongly preferred. This is in line with previous insights that revealed that participants want to have greater control over the entire data research process [14,27,29,40]. Thus, our findings reinforce the current understanding about the importance of personal control over health data sharing for research [8,17,18]. Examples are demanding that researchers should ask for consent each time data are used as well as make it possible for participants to decide which researchers can use particular types of data and for which research projects.

Yet, participants value personal control far more than simply and only the practice of giving up-front informed specific consent. Our findings highlight that personal control should be understood and can be put to practice more broadly than the specific forms of control with which we are familiar. In addition to traditional and conventional ethical requirements like consent, personal control can comprise less conventional means to empower participants [22,23,29]. They can be given opportunities to audit who has used their data as well as how their data have been used. Nevertheless, strengthening personal control is far less important as people are more favorable to health data sharing. This emphasizes current insights about the importance of conditions that build trust in data-intensive health research [8,14,21,41]. Our findings support the hypothesis that ethics requirements and governance policies establish the trustworthiness of research organizations and data-sharing practices [5,6,17,18]. Hence, they are crucial to warrant greater trust by patients and publics [6,14,28].

A second type of policies and measures that were deemed important is located at the collective level of governance. Current insights recognize that governance policies and measures strengthen transparency and engender responsible conduct, which are important for accountability and trustworthiness [8,14,18,29,42,43]. Our findings point out that governance policies and measures are considered valuable since they strengthen possibilities for participants to exercise control on health data sharing. Governance raises the level of control over health data research to that of the collective. In large-scale health data research, this leap facilitates building transparency and trustworthiness beyond the limitations faced by individual research participants.

In addition to corroborating previous insights, our findings put more flesh on the bones of what governance policies and measures could look like. We highlight that this type of governance fulfills a performative function since it shapes a clear and consistent framework on which trust can be built. Having such a framework clarifies the consequences of data misuse and neglect of responsibility. This exemplifies that participants feel the need for hard-and-fast safeguards, measures, and policies. Sanctions can serve to demarcate the boundaries of permissibility in health data research, as the purposes to which data are put are called into question and uncertainty prevails [8,18].

Our findings underline how relevant awareness of patient and public involvement is for willingness to partake in data-intensive health research. Expanding patient and public involvement roles in governance particularly requires attention. This substantiates suitable and meaningful patient and public involvement as an important way of increasing trust, since it fosters greater mutual understanding and a more open research process [8,12,27,44]. See Table 7 for an overview of the main points and key takeaways from the discussion.

**Table 7:** Table summarizing the main points and key takeaways of the discussion.

What was known before	What this study adds	Implications for practice
Protecting patients’ privacy and the confidentiality and security of personal health data are important concerns in patients’ attitudes to data sharing and linkage [5,8,14,19,20].	Respondents *prefer* anonymous data sharing, closely followed by the option of pseudonymous data sharing.	It is important to explore possibilities for utilizing pseudonymous data sharing in governance policies.
Data-intensive health research’s status as a common good is widespread, which contributes to gaining and retaining support [8,15,22,23].	A research (question)’s relevance is more important than restricting data access to not-for-profit researchers or organizations only.	Warranties that can explicate public benefit and contribute to maintaining support should be developed further as an integral part of governance.
Data use by commercial parties seeking profit, like pharmaceutical companies, diminishes its perceived public benefit [14,18,19].	Shared health data perceived to be used by private parties for the purpose of commercial gain is detrimental to patients’ and publics’ support and willingness to partake in data-intensive health research.	Establishing relevance is crucial to public-private cooperation in research, yet more insight is needed into how such relevance can be strengthened and secured.
Research participants want to be facilitated to have greater control over the process of health data sharing and research [8,17,18].	Respondents prefer being enabled to exercise various specific forms of individual-level, personal control.	Research participants should be able to decide who can gain access, to which types of data, and for which endeavors.
Personal control plays an important role in patients’ and publics’ views on how governance should be shaped [20,22,26,27].	Personal control should go beyond conventional roles of research participants, such as giving consent.	Research participants should be empowered via unconventional and innovative tools, such as participant-initiated data auditing.
Beyond personal control, patients and publics prefer governance arrangements to garner trust in data-intensive health research [5,6,17,18].	People in favor of health data sharing require less means of exercising personal control.	Establishing trustworthiness of research organizations and data sharing practices should be a central goal of designing governance.
Governance strengthens transparency and engenders responsible conduct, which are important for accountability and trustworthiness [8,14,18,29,42,43].	Governance measures are considered valuable since they strengthen possibilities to exercise collective control on health data sharing.	Raising control to a collective level allows going beyond the limits of individual control in large-scale health data research.
Participants experience uncertainty about what are permissible purposes for data use and require hard-and-fast safeguards [8,18].	Shaping a clear and consistent framework of consequences for data misuse and neglect of responsibility is crucial in governance.	Governance should create and demarcate normative boundaries, backed by repercussions such as sanctions when not respected.
Meaningful patient and public involvement is important to foster mutual understanding and a more open research process [8,12,27,44].	Patient and public involvement in governance contributes to willingness to partake in research.	Patient and public involvement should be expanded and assigned various roles as part of governance.

Comparing individual-level, personal control to control at the collective level of governance, the latter stands out. Implementing such policies and measures facilitates establishing clear-cut governance frameworks, which can merit conditions that need to be met for patients and publics to support data-intensive health research. Various policies and measures need to be pursued to ensure trust in proper purpose, use, and protection of health data. Policy requirements that safeguard the security of databases should be developed. Measures to impose sanctions for data misuse need to be implemented. Finally, reliability checks for researchers should be incorporated.

### Strengths and Weaknesses

The purposive sampling strategy that we employed precludes straightforward generalization of our findings. This means that we must be careful in interpreting the findings in the context of a wider population. The results of this study are likely to represent patients’ and publics’ preferences that tend to patient advocacy since distribution was facilitated by the European Heart Network. Additionally, the study population overrepresented older age groups, men, and residents of the Netherlands. Most respondents had completed vocational education or possessed professional qualifications. Moreover, they identified as patients. Yet, these characteristics are in line with what is expected from the population of patients and publics involved with cardiovascular diseases from which we sampled. Our sample and findings seem to be representative of this group. Additionally, as education level was significantly associated with several dependent variables, the role of this background variable in the results needs to be stated. Further research could benefit from a systematic, probability, stratified or cohort sampling approach. Doing so would forestall limitations of population diversity and facilitate generalization to a broader population of patients and publics. These factors may have contributed to more positive tendencies in preferences and slightly stronger associations between variables. However, they were unlikely to have strongly distorted the findings, such as changes in positive versus negative distributions, or the direction of associations.

We should be cautious about qualitative interpretation of our results. The quantitative methods we employed only provide limited means of doing so. Future research on patients’ and publics’ preferences for data-intensive health research governance could benefit from employing qualitative methods. Conducting interviews or focus groups facilitates painting a richer picture of the motivations and reasons for the preferences we found. Mixed method approaches such as sequential explanatory designs could provide interesting insights by triangulating quantitative and qualitative methodologies in this field of inquiry.

### Conclusions

Policies and measures are crucial for governing data-intensive health research and building trust. The findings of this study point out that greater attention should be directed to patients’ and publics’ preferences for control at the collective level of governance than has hitherto been recognized. This confirms the slow but steady shift to understanding conditions for support of data-intensive health research to operationalize governance policies and measures. Our findings further entrench that governance functions by building on conditions for support and furthers trustworthiness of data-intensive health research. This resonates with preparatory developments that are part of establishing the European Health Data Space [10,11].

We recommend that future research explores patients’ and publics’ meaning-making and interpretation of control at the collective level of governance for data-intensive health research. Future research needs to address how specific varieties of governance policies and measures can be shaped in practice in accordance with conditions for support of health data sharing and research. Sanctioning data misuse is one policy that requires exploration in greater detail. We described data misuse as attempting to trace anonymous health data back to one’s identity, yet it remains opaque what patients and publics exactly see as data misuse. This is a critical topic for policy making that needs to be addressed. What types of sanctions for data misuse would be regarded as appropriate and required to warrant trust needs to be studied further. The development of reliability checks for researchers and under what conditions an independent evaluation committee should be pursued need further study as well. Attaining insight in the views of research participants, publics, and professionals is crucial to establish provisional fixed points for the governance of data-intensive health research.

## Appendix

Multimedia Appendix 1 Questionnaire to capture patients’ and publics' preferences for data-intensive health research governance.

Multimedia Appendix 2 Results tables.

## Abbreviations

IMI2: Innovative Medicines Initiative 2 Joint Undertaking
EFPIA: European Federation of Pharmaceutical Industries and Associations

## References

[1] Scheibner J, Ienca M, Kechagia S, Troncoso-Pastoriza JR, Raisaro JL, Hubaux J, Fellay J, Vayena E (2020). Data protection and ethics requirements for multisite research with health data: a comparative examination of legislative governance frameworks and the role of data protection technologies. J Law Biosci.

[2] Kaye J, Terry SF, Juengst E, Coy S, Harris JR, Chalmers D, Dove ES, Budin-Ljøsne I, Adebamowo C, Ogbe E, Bezuidenhout L, Morrison M, Minion JT, Murtagh MJ, Minari J, Teare H, Isasi R, Kato K, Rial-Sebbag E, Marshall P, Koenig B, Cambon-Thomsen A (2018). Including all voices in international data-sharing governance. Hum Genomics.

[3] Kalkman S, Mostert M, Gerlinger C, van Delden JJM, van Thiel GJMW (2019). Responsible data sharing in international health research: a systematic review of principles and norms. BMC Med Ethics.

[4] Kalkman S, Mostert M, Udo-Beauvisage N, van Delden JJ, van Thiel GJ (2019). Responsible data sharing in a big data-driven translational research platform: lessons learned. BMC Med Inform Decis Mak.

[5] Kalkman S, van Delden J, Banerjee A, Tyl B, Mostert M, van Thiel G (2022). Patients' and public views and attitudes towards the sharing of health data for research: a narrative review of the empirical evidence. J Med Ethics.

[6] Carter P, Laurie GT, Dixon-Woods M (2015). The social licence for research: why care.data ran into trouble. J Med Ethics.

[7] Muller SHA, Kalkman S, van Thiel GJMW, Mostert M, van Delden JJM (2021). The social licence for data-intensive health research: towards co-creation, public value and trust. BMC Med Ethics.

[8] Aitken M, de St Jorre J, Pagliari C, Jepson R, Cunningham-Burley S (2016). Public responses to the sharing and linkage of health data for research purposes: a systematic review and thematic synthesis of qualitative studies. BMC Med Ethics.

[9] (2020). Communication from the Commission to the European Parliament, the Council, the European Economic and Social Committee and the Committee of the Regions: A European Strategy for Data. European Commission.

[10] (2021). European Health Union: Commission publishes open public consultation on the European Health Data Space. European Commission.

[11] Hansen J, Wilson P, Verhoeven E, Kroneman M, Kirwan M, Verheij R (2021). Assessment of the EU Member States' rules on health data in the light of GDPR. Publications Office of the European Union.

[12] Erikainen S, Friesen P, Rand L, Jongsma K, Dunn M, Sorbie A, McCoy M, Bell J, Burgess M, Chen H, Chico V, Cunningham-Burley S, Darbyshire J, Dawson R, Evans A, Fahy N, Finlay T, Frith L, Goldenberg A, Hinton L, Hoppe N, Hughes N, Koenig B, Lignou S, McGowan M, Parker M, Prainsack B, Shabani M, Staunton C, Thompson R, Varnai K, Vayena E, Williams O, Williamson M, Chan S, Sheehan M (2020). Public involvement in the governance of population-level biomedical research: unresolved questions and future directions. J Med Ethics.

[13] Felt U, Fochler M (2010). Machineries for making publics: inscribing and de-scribing publics in public engagement. Minerva.

[14] Stockdale J, Cassell J, Ford E (2018). "Giving something back": A systematic review and ethical enquiry into public views on the use of patient data for research in the United Kingdom and the Republic of Ireland. Wellcome Open Res.

[15] Eloranta K, Auvinen A (2015). Population attitudes towards research use of health care registries: a population-based survey in Finland. BMC Med Ethics.

[16] Shah N, Coathup V, Teare H, Forgie I, Giordano GN, Hansen TH, Groeneveld L, Hudson M, Pearson E, Ruetten H, Kaye J (2019). Sharing data for future research-engaging participants' views about data governance beyond the original project: a DIRECT Study. Genet Med.

[17] Hill EM, Turner EL, Martin RM, Donovan JL (2013). "Let's get the best quality research we can": public awareness and acceptance of consent to use existing data in health research: a systematic review and qualitative study. BMC Med Res Methodol.

[18] Howe N, Giles E, Newbury-Birch D, McColl E (2018). Systematic review of participants' attitudes towards data sharing: a thematic synthesis. J Health Serv Res Policy.

[19] Garrison NA, Sathe NA, Antommaria AHM, Holm IA, Sanderson SC, Smith ME, McPheeters ML, Clayton EW (2016). A systematic literature review of individuals' perspectives on broad consent and data sharing in the United States. Genet Med.

[20] King T, Brankovic L, Gillard P (2012). Perspectives of Australian adults about protecting the privacy of their health information in statistical databases. Int J Med Inform.

[21] Mählmann L, Schee Gen Halfmann S, von Wyl A, Brand A (2017). Attitudes towards personal genomics and sharing of genetic data among older Swiss adults: a qualitative study. Public Health Genomics.

[22] Willison DJ, Swinton M, Schwartz L, Abelson J, Charles C, Northrup D, Cheng J, Thabane L (2008). Alternatives to project-specific consent for access to personal information for health research: insights from a public dialogue. BMC Med Ethics.

[23] Clerkin P, Buckley BS, Murphy AW, MacFarlane AE (2013). Patients' views about the use of their personal information from general practice medical records in health research: a qualitative study in Ireland. Fam Pract.

[24] Sethi N, Laurie GT (2013). Delivering proportionate governance in the era of eHealth: Making linkage and privacy work together. Med Law Int.

[25] Krahe M, Milligan E, Reilly S (2019). Personal health information in research: Perceived risk, trustworthiness and opinions from patients attending a tertiary healthcare facility. J Biomed Inform.

[26] Buckley BS, Murphy AW, MacFarlane AE (2011). Public attitudes to the use in research of personal health information from general practitioners' records: a survey of the Irish general public. J Med Ethics.

[27] Robling MR, Hood K, Houston H, Pill R, Fay J, Evans HM (2004). Public attitudes towards the use of primary care patient record data in medical research without consent: a qualitative study. J Med Ethics.

[28] Boers SN, van Delden JJM, Bredenoord AL (2015). Broad consent is consent for governance. Am J Bioeth.

[29] Damschroder LJ, Pritts JL, Neblo MA, Kalarickal RJ, Creswell JW, Hayward RA (2007). Patients, privacy and trust: patients' willingness to allow researchers to access their medical records. Soc Sci Med.

[30] Jamieson S (2004). Likert scales: how to (ab)use them. Med Educ.

[31] Pett MA (2016). Nonparametric Statistics for Health Care Research: Statistics for Small Samples and Unusual Distributions.

[32] Knapp TR (1990). Treating ordinal scales as interval scales: an attempt to resolve the controversy. Nurs Res.

[33] Sullivan GM, Artino AR (2013). Analyzing and interpreting data from likert-type scales. J Grad Med Educ.

[34] Tabachnick BG, Fidell LS (2013). Using Multivariate Statistics.

[35] Leeuw E, Hox J, Lavrakas P (2008). Missing Data. Encyclopedia of Survey Research Methods.

[36] Spencer K, Sanders C, Whitley EA, Lund D, Kaye J, Dixon WG (2016). Patient perspectives on sharing anonymized personal health data using a digital system for dynamic consent and research feedback: a qualitative study. J Med Internet Res.

[37] Aitken M, McAteer G, Davidson S, Frostick C, Cunningham-Burley S (2018). Public preferences regarding data linkage for health research: a discrete choice experiment. Int J Popul Data Sci.

[38] van Veen E (2018). Observational health research in Europe: understanding the General Data Protection Regulation and underlying debate. Eur J Cancer.

[39] Mostert M, Bredenoord AL, Biesaart MCIH, van Delden JJM (2016). Big Data in medical research and EU data protection law: challenges to the consent or anonymise approach. Eur J Hum Genet.

[40] Shabani M, Bezuidenhout L, Borry P (2014). Attitudes of research participants and the general public towards genomic data sharing: a systematic literature review. Expert Rev Mol Diagn.

[41] Aitken M, Cunningham-Burley S, Pagliari C (2016). Moving from trust to trustworthiness: Experiences of public engagement in the Scottish Health Informatics Programme. Sci Public Policy.

[42] Teng J, Bentley C, Burgess MM, O'Doherty KC, McGrail KM (2019). Sharing linked data sets for research: results from a deliberative public engagement event in British Columbia, Canada. Int J Popul Data Sci.

[43] Paprica PA, de Melo MN, Schull MJ (2019). Social licence and the general public's attitudes toward research based on linked administrative health data: a qualitative study. CMAJ Open.

[44] Nair K, Willison D, Holbrook A, Keshavjee K (2004). Patients' consent preferences regarding the use of their health information for research purposes: a qualitative study. J Health Serv Res Policy.

