# Supplementary material for: Patients’ and Publics’ Preferences for Data-Intensive Health Research Governance: Survey Study
Source: JMIR Hum Factors. 2022 Sep 7;9(3):e36797. doi: 10.2196/36797 (PMC9494211; doi:10.2196/36797)

**Multimedia Appendix 1: Questionnaire to Capture Patients’ and Publics’ Preferences for Data-Intensive Health Research Governance**


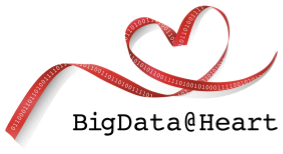


**Your opinion about sharing health data for scientific health research**

Thank you for taking part in this survey. The survey is about your opinion on sharing health data for scientific research. Health data will only be collected if you have given your consent or if the data is completely anonymous and if you are participating in the medical research. Which data is collected differs per study. For example, this may include:

- General information such as age and gender
- Data about the disease, the treatment and its effect
- Laboratory results such as blood and urine tests
- The results of imaging tests, such as X-rays, MRI and CT scans
- Data collected via wearables. For example, the sleep rhythm or the number of steps taken are tracked by a smartwatch
- Body tissue/material stored for research, such as DNA or tumour tissue

By exchanging health data between researchers, more data becomes available and new connections can be found. By combining the data from different databases researchers believe they can gain better insights into, for example, diagnosis and treatments of several diseases and conditions. Support and cooperation of patients and citizens is essential to achieve this goal. It is therefore important to find out what patients and citizens think of the (large-scale) combining and sharing of data for scientific health research. That is what this survey is about.

This survey study is part of a European project called BigData@Heart. The purpose of the project is to create digital opportunities for large-scale research in cardiovascular health through the sharing and collation of health data. Given the large amount of health data, this is also referred to as "Big Data". (https://bigdata-heart.eu). The researchers who prepared this survey work as researchers at the University Medical Center Utrecht in the Netherlands. The European Heart Network is one of the partners in the project. For more information about this project, please visit [https://bigdata-heart.eu](https://bigdata-heart.eu/)

If you have any questions regarding this survey, please contact Dr Ghislaine van Thiel at

g.j.m.w.vanthiel@umcutrecht.nl

This survey consists of 17 questions and will take you roughly 10 minutes to fill in. By responding to the survey, you will consent to your answers being used in a scientific research study by our team at the University Medical Center Utrecht. We aim to publish our findings in a scientific journal. Unless you decide to leave your email address at the end of the questionnaire, you will remain anonymous to us.

*This project has received funding from the Innovative Medicines Initiative 2 Joint Undertaking under grant agreement No 115966. This Joint Undertaking receives support from the European Union’s Horizon 2020 research and innovation programme and EFPIA.*

**Your views on data sharing**

We would like to know how you feel, generally speaking, about the sharing of your health data for scientific health research purposes.

**1. In general, how do you feel about sharing your health data for health research?**

| Strongly oppose | Somewhat oppose | Neutral | Somewhat favour | Strongly favour | I don’t know |
| --- | --- | --- | --- | --- | --- |
| O | O | O | O | O | O |

**2. With regard to anonymity of data used for health research: which of the following statements best reflects your opinion?**

O It is a requirement for me to only share my health data anonymously (without names or other identifying details)

O I prefer to share my data anonymously (without names or other identifying details)

O I prefer to share my data pseudononimised (meaning data will contain a code but not a name or other data that can identify you. The code and a key can identify you. The key is stored securely in the research facility).

O I allow sharing of my data including my name or other personal information

**3. How important is it that you can decide for which research projects your health data are shared?**

| Not at all important | Of low importance | Slightly important | Neutral | Moderately important | Very important | Extremely important | I don’t know |
| --- | --- | --- | --- | --- | --- | --- | --- |
| O | O | O | O | O | O | O | O |

Optional: Explain your answer

**4. How important is it that you are informed about the research projects for which your health data is shared?**

| Not at all important | Of low importance | Slightly important | Neutral | Moderately important | Very important | Extremely important | I don’t know |
| --- | --- | --- | --- | --- | --- | --- | --- |
| O | O | O | O | O | O | O | O |

Optional: Explain your answer

**5. How important is it that you can decide for yourself which researchers/organisations your health data is shared with?**

| Not at all important | Of low importance | Slightly important | Neutral | Moderately important | Very important | Extremely important | I don’t know |
| --- | --- | --- | --- | --- | --- | --- | --- |
| O | O | O | O | O | O | O | O |

Optional: Explain your answer

**6. How important is it that you can choose which health data is shared and which is not?**

| Not at all important | Of low importance | Slightly important | Neutral | Moderately important | Very important | Extremely important | I don’t know |
| --- | --- | --- | --- | --- | --- | --- | --- |
| O | O | O | O | O | O | O | O |

Optional: Explain your answer

**7. Which researchers do you think should have access to your health data?**

O All researchers/organisations who have a relevant research question (e.g. for scientific research on diagnostics and/or treatments)

O Only researchers from governments or not-for-profit organisations (not-for-profit organisations are organisations without a profit motive, for example universities, hospitals, or research institutions)

O All researchers irrespective of the scientific or social relevance of the research (this includes pharmaceutical companies and companies developing medical equipment)

O All, not only researchers, but also persons such as representatives of patient and citizen associations, or scientific journalists

**Your views on health data sharing policies**

Data sharing policies refer to one or more rules that researchers must follow when using or sharing health data from health databases. This type of governance often addresses things like who will be responsible for managing your information and what measures are in place to protect your interests. We would like to know what you think is important in health data sharing policies.

**8. Please indicate how important the following factors are when your data are shared with other researchers outside of the original study?**

|  | Not at all important | Of little importance | Somewhat important | Neutraal | Fairly important | Very important | Extremely important | I don’t know |
| --- | --- | --- | --- | --- | --- | --- | --- | --- |
| The database is highly secure (i.e. it is very difficult to get into the location where the health data is stored) | O | O | O | O | O | O |  |  |
| I can have my health data deleted at any time | O | O | O | O | O | O |  |  |
| I can decide on the conditions under which health data can be shared (e.g. limitation of international data sharing or commercial use) | O | O | O | O | O | O |  |  |
| Before researchers are given access to health data, they must be checked for reliability. This means ensuring the proper purpose and protection of the health data. | O | O | O | O | O | O |  |  |

**9. Please select your top three (3) agreements and rules that are the most important to you:**

O Requests for access to health data should be evaluated by an *independent* (data access) committee that has no interest in the research

O Researchers should *ask for consent* of *of the patients/citizens from whom these data originate* each time their health data will be used

O Researchers should *notify* patients/citizens that their health data will be re-used

O Researchers should obtain approval from representatives on behalf of patients/citizens to use their health data

O Researchers should only be allowed to use the health data for a pre-approved period of time. After this period, the health data can no longer be used

O If health data is misused, those concerned must be subject to sanctions. Misuse includes attempting to trace anonymous health data back to your identity

O Researchers should only inform patients/citizens about the results of the research studies for which their health data was used

**Your opinion on patient participation?**

In health research, patient participation is often defined as research conducted by talking 'to' patients rather than talking 'about' patients. We would like to know what your opinion is on involving patients when health data is shared.

**10. Have you ever heard of patients being involved in health research? For example, participation in review committees or sounding board groups?**

O Yes
O No
O I don’t know

**11. Have you ever participated in activities that could be considered patient involvement in health research? For example, participation in review committees or sounding board groups?**

O Yes
O No
O I don’t know

**12. In your opinion, how important is patient involvement for the following aspects of health research?**

| Patients and/or citizens should be involved in | Extremely important | Fairly important | Not important or unimportant | Fairly unimportant | Not important at all | I don’t know |
| --- | --- | --- | --- | --- | --- | --- |
| Making choices about which research questions are relevant in medical science | O | O | O | O | O | O |
| Making choices about how to conduct research that uses health data | O | O | O | O | O | O |
| Making choices about providing information and consent regarding the use of health data. (i.e. data may/may not be used with/without explicit consent) | O | O | O | O | O | O |
| Evaluating requests to share health data. (i.e. as a member of a review committee, a patient can determine who has access to the health data and for what purpose) | O | O | O | O | O | O |
| The dissemination of research results (i.e. choosing journals, websites, etc. in which to publish the results of the research) | O | O | O | O | O | O |

And finally, please tell us something about yourself (not mandatory)

**13. What is your gender?**

O Female

O Male
O Other
O Prefer not to say

**14. What is your age category? (years)**

O 18-30

O 31-40
O 41-50

O 51-60

O 61-70
O 71+
O Prefer not to say

**15. Which country do you live in?**

[Drop down menu of nationalities/countries – list does not need to be translated]

**16. What is your highest level of education?**

O Less than secondary / high school
O Secondary / high school
O Vocational/professional qualifications

O Bachelor’s degree
O Master’s degree
O Postgraduate degree
O Other
O Prefer not to say

**17. Do you see yourself as someone with a heart or vascular disease?**

O Yes
O No
O Prefer not to say

**Is there anything you would like to add to the information in this survey?**


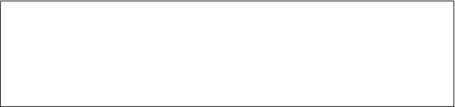


We thank you for taking part in this survey! If you wish to receive updates about this study please leave your e-mail address below:


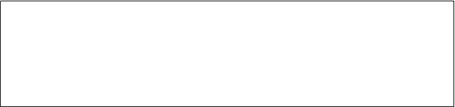

Supplement: Multimedia Appendix 1 [file humanfactors_v9i3e36797_app1.doc]
