# Supplementary material for: Patients’ and Publics’ Preferences for Data-Intensive Health Research Governance: Survey Study
Source: JMIR Hum Factors. 2022 Sep 7;9(3):e36797. doi: 10.2196/36797 (PMC9494211; doi:10.2196/36797)
Supplement: Multimedia Appendix 2 [file humanfactors_v9i3e36797_app2.doc]

**Multimedia Appendix 2: Results Tables**

Table S1. Descriptive statistics and frequencies for general views on health data sharing conditions.

|  | (1) % Strongly op­pose *(n)*^a^ | (2) % Some­what oppose *(n)* | | (3) % Neutral *(n)* | (4) % Some­what favor *(n)* | (5) % Strongly favor *(n)* | *Md* | IQR |
| --- | --- | --- | --- | --- | --- | --- | --- | --- |
| In general, how do you feel about sharing your health data for health research? | 1 *(10)* | 3.5 *(34)* | | 9.1 *(89)* | 23.8 *(233)* | 62.7 *(615)* | 5 | 4-5 |
|  | (1) % Highly unimportant *(n)* | (2) % Fairly unimportant *(n)* | | (3) % Neither important nor unimportant *(n)* | (4) % Mod­erately im­portant *(n)* | (5) % Highly important *(n)* |  |  |
| How important is it that you can decide for which research projects your health data are shared? | 23.1 *(226)* | 7.4 *(73)* | | 14.5 *(142)* | 19.7 *(193)* | 35.3 *(346)* | 5 | 2-5 |
| How important is it that you are informed about the research projects for which your health data is shared? | 8.7 *(85)* | 7.7 *(76)* | | 11.2 *(110)* | 25.7 *(252)* | 46.7 *(458)* | 5 | 3-5 |
| How important is it that you can decide for yourself which research­ers/organizations your health data is shared with? | 17.7 *(173)* | 8.5 *(83)* | | 16.4 *(160)* | 18.6 *(182)* | 38.9 *(380)* | 5 | 2-5 |
| How important is it that you can choose which health data is shared and which is not? | 26.7 *(261)* | 6.2 *(61)* | | 16.5 *(161)* | 17.3 *(169)* | 33.3 *(325)* | 5 | 1-5 |
|  | | |  | | % *(n)* | | *Mo* | |
| *Regarding anonymity of data used for health research: which of the following statements best reflects your opinion?* | | | | |  | | 2 | |
| 1. It is a requirement for me to only share my health data anonymously | | | | | 22.9 *(226)* | |  |  |
| 2. I prefer to share my data anonymously | | | | | 33.3 *(328)* | |  |  |
| 3. I prefer to share my data pseudonymously | | | | | 26.1 *(257)* | |  |  |
| 4. I allow sharing of my data including my name or other personal infor­mation | | | | | 17.7 *(174)* | |  |  |
| *Which researchers do you think should have access to your health data?* | | | | |  | | 1 | |
| 1. All researchers/organizations who have a relevant research question | | | | | 43 *(423)* | |  |  |
| 2. Only researchers from governments or not-for-profit organizations | | | | | 43 *(423)* | |  |  |
| 3. All researchers irrespective of the scientific or social relevance of the re­search | | | | | 10 *(98)* | |  |  |
| 4. All, not only researchers, but also persons such as representatives of pa­tient and citizen associations, or scientific journalists | | | | | 4 *(39)* | |  |  |
| ^a^Percentages given are valid percentages; *n* varies per variable. | | | | | | | | |

Table S2. Descriptive statistics and frequencies for health data sharing policies and governance measures.

|  | (1) % Highly unimportant *(n)*^a^ | (2) % Fairly unimportant *(n)* | (3) % Neither important nor unimportant *(n)* | (4) % Moder­ately im­portant *(n)* | (5) % Highly im­portant *(n)* | *Md* | IQR |
| --- | --- | --- | --- | --- | --- | --- | --- |
| *What are important factors when your data are shared with outside of the orig­inal study?* |  | | | | |  | |
| The database is highly secure | 1.6 *(16)* | 3 *(29)* | 4.9 *(48)* | 9.8 *(96)* | 80.6 *(787)* | 5 | 5-5 |
| I can have my health data deleted at any time | 5.5 *(53)* | 4.3 *(42)* | 13.1 *(127)* | 16.9 *(161)* | 60.6 *(589)* | 5 | 4-5 |
| I can decide on the con­ditions under which health data can be shared | 7.8 *(76)* | 4.7 *(46)* | 12.2 *(118)* | 19.7 *(191)* | 55.5 *(538)* | 5 | 4-5 |
| Before researchers are given access to health data, they must be checked for reliability | 0.9 *(9)* | 2.3 *(22)* | 2 *(19)* | 11.6 *(112)* | 83.2 *(805)* | 5 | 5-5 |
|  | | | | | % *(n)* | *Mo* | |
| *Please select the top three agreements and rules that are the most important to you.*^b^ | | | | | | 6 | |
| 1. Requests for access to health data should be evaluated by an independent (data access) com­mittee | | | | | 22.4 *(607)* |  | |
| 2. Researchers should ask for consent of the patients/citizens from whom these data originate each time their health data will be used | | | | | 12 *(324)* |  | |
| 3. Researchers should notify patients/citizens that their health data will be re-used | | | | | 13.2 *(358)* |  | |
| 4. Researchers should obtain approval from representatives on behalf of patients/citizens to use their health data | | | | | 8.9 *(242)* |  | |
| 5. Researchers should only be allowed to use the health data for a pre-approved time period. Af­ter this period, the health data can no longer be used | | | | | 12.2 *(331)* |  | |
| 6. If health data is misused, those concerned must be subject to sanctions. | | | | | 23.5 *(637)* |  | |
| 7. Researchers should only inform patients/citizens about the results of the research studies for which their health data was used | | | | | 7.6 *(209)* |  | |
| ^a^Percentages given are valid percentages; *n* varies per variable.  ^b^The total *n* of 2,708 for this variable resulted from summing the three options selected by respondents. | | | | | | | |

Table S3. Spearman rank order correlations (ρ) between general willingness to share health data and data sharing policies.

| **Spearman rank order correlation** | **1.^a^ *(P)*** | **2. *(P)*** | **3. *(P)*** | **4. *(P)*** | **5. *(P)*** |
| --- | --- | --- | --- | --- | --- |
| **1.** In general, how do you feel about sharing your health data for health re­search? | - |  |  |  |  |
| **2.** The database is highly secure | 0.045  (.164) | - |  |  |  |
| **3.** I can have my health data deleted at any time | -0.118  (<.001) | 0.340  (<.001) | - |  |  |
| **4.** I can decide on the conditions under which health data can be shared | -0.173  (<.001) | 0.352  (<.001) | 0.524  (<.001) | - |  |
| **5.** Before researchers are given access to health data, they must be checked for reliability | 0.027  (.410) | 0.409  (<.001) | 0.355  (<.001) | 0.442  (<.001) | - |
| ^a^The column numbers correspond to the row number provided for each variable. | | | | | |

Table S4. Spearman rank order correlations (ρ) between preference to decide on conditions under which health data are shared and health data sharing policies.

| **Spearman rank order correlation** | **1.^a^ *(P)*** | **2. *(P)*** | **3. *(P)*** | **4. *(P)*** |
| --- | --- | --- | --- | --- |
| **1.** I can decide on the conditions under which health data can be shared | - |  |  |  |
| **2.** The database is highly secure | 0.352  (<.001) | - |  |  |
| **3.** I can have my health data deleted at any time | 0.524  (<.001) | 0.340  (<.001) | - |  |
| **4.** Before researchers are given access to health data, they must be checked for reliability | 0.442  (<.001) | 0.409  (<.001) | 0.355  (<.001) | - |
| ^a^The column numbers correspond to the row number provided for each variable. | | | | |

Table S5. Descriptive statistics and frequencies for patient and public involvement in health data

sharing.

|  | (1) % Not at all important *(n)*^a^ | (2) % Slightly im­portant *(n)* | | (3) % Neu­tral *(n)* | | (4) % Fairly important *(n)* | | (5) % Ex­tremely im­portant *(n)* | *Md* | IQR |
| --- | --- | --- | --- | --- | --- | --- | --- | --- | --- | --- |
| *Patients and/or citizens should be involved in …* |  | | | | | | | |  | |
| Making choices about which re­search questions are relevant in medical science | 10.7 *(100)* | 14.3 *(133)* | | 19 *(177)* | | 38.8 *(361)* | | 17.2 *(160)* | 4 | 2-4 |
| Making choices about how to conduct research that uses health data | 12.8 *(119)* | 14.6 *(136)* | | 21.4 *(199)* | | 35.4 *(330)* | | 15.8 *(147)* | 4 | 2-4 |
| Making choices about providing information and consent regard­ing the use of health data | 3.9 *(37)* | 8.5 *(81)* | | 15.9 *(151)* | | 40.3 *(383)* | | 31.4 *(299)* | 4 | 3-5 |
| Evaluating requests to share health data | 7.7 *(71)* | 10.7 *(99)* | | 20.8 *(192)* | | 39.3 *(362)* | | 21.4 *(197)* | 4 | 3-4 |
| The dissemination of research results | 12.2 *(116)* | 12.6 *(120)* | | 24 *(228)* | | 30.8 *(292)* | | 20.3 *(193)* | 4 | 3-4 |
|  |  | | (1) %Yes *(n)* | | (2) % No *(n)* | | (3) I don’t know | | *Mo* | |
| Have you ever heard of patients being involved in health re­search? | | | 47.2 *(466)* | | 44.7 *(441)* | | 8.1 *(80)* | | 1 | |
| Have you ever participated in activities that could be considered patient involvement in health research? | | | 21.7 *(214)* | | 75.2 *(742)* | | 3.1 *(31)* | | 2 | |
| ^a^Percentages given are valid percentages; *n* varies per variable. | | | | | | | | | | |

Table S6. Spearman rank order correlations (ρ) between general willingness to share health data and patient and public involvement roles in health data research.

| **Spearman rank order correlation** | **1.^a^ *(P)*** | **2. *(P)*** | **3. *(P)*** | **4. *(P)*** | **5. *(P)*** | **6. *(P)*** |
| --- | --- | --- | --- | --- | --- | --- |
| **1.** In general, how do you feel about shar­ing your health data for health research? | - |  |  |  |  |  |
| **2.** Making choices about which research questions are relevant in medical science | -0.038  (.242) | - |  |  |  |  |
| **3.** Making choices about how to conduct research that uses health data | -0.065  (.050) | 0.698  (<.001) | - |  |  |  |
| **4.** Making choices about providing infor­mation and consent regarding the use of health data | -0.113  (<.001) | 0.425  (<.001) | 0.494  (<.001) | - |  |  |
| **5.** Evaluating requests to share health data | -0.102  (.002) | 0.479  (<.001) | 0.507  (<.001) | 0.609  (<.001) | - |  |
| **6.** The dissemination of research results | -0.071  (.030) | 0.498  (<.001) | 0.518  (<.001) | 0.386  (<.001) | 0.482 (<.001) | - |
| ^a^The column numbers correspond to the row number provided for each variable. | | | | | | |

Table S7. Mann-Whitney U tests for awareness of patient and public involvement and importance of roles.

| **Mann-Whitney U test** | *Md (n)* | *Md (n)* | Mean Rank | | *U* | *z* | *P* value |
| --- | --- | --- | --- | --- | --- | --- | --- |
|  | **Yes** | **No** | **Yes** | **No** |  |  |  |
| Making choices about which research ques­tions are relevant in medical science | 4 (442) | 4 (413) | 466 | 387 | 74336 | -4.89 | <.001 |
| Making choices about how to conduct re­search that uses health data | 4 (446) | 3 (412) | 460 | 396 | 78079 | -3.94 | <.001 |
| Making choices about providing information and consent regarding the use of health data | 4 (459) | 4 (417) | 457 | 419 | 87388 | -2.35 | .019 |
| Evaluating requests to share health data | 4 (446) | 4 (406) | 443 | 408 | 83211 | -2.13 | .033 |
| The dissemination of research results | 4 (454) | 4 (420) | 445 | 430 | 92009 | -0.92 | .358 |
